# Caffeine protects against stress-induced murine depression through activation of PPARγC1α-mediated restoration of the kynurenine pathway in the skeletal muscle

**DOI:** 10.1038/s41598-021-86659-4

**Published:** 2021-03-31

**Authors:** Chongye Fang, Shuhei Hayashi, Xiaocui Du, Xianbin Cai, Bin Deng, Hongmei Zheng, Satoshi Ishido, Hiroko Tsutsui, Jun Sheng

**Affiliations:** 1 Yunnan Research Center for Advanced Tea Processing, College of Pu-erh Tea, Yunnan Agricultural University, Kunming, 650201 China; 2 Department of Internal Medicine, Hyogo College of Medicine, 1-1 Mukogawa-cho, Nishinomiya, Hyogo 663-8501 Japan; 3 Department of Gastroenterology, First Affiliated Hospital of Shantou University Medical College, Shantou, 515041 Guangdong China; 4 Department of Microbiology, Hyogo College of Medicine, 1-1 Mukogawa-cho, Nishinomiya, Hyogo 663-8501 Japan; 5 Yunnan Rural Science and Technology Service Center, Kunming, Yunnan China; 6 Key Laboratory of Pu-erh Tea Science, The Ministry of Education, Yunnan Agricultural University, Kunming, 650201 China

**Keywords:** Diseases of the nervous system, Emotion

## Abstract

Exercise prevents depression through peroxisome proliferator-activated receptor-gamma coactivator 1α (PGC-1α)-mediated activation of a particular branch of the kynurenine pathway. From kynurenine (KYN), two independent metabolic pathways produce neurofunctionally different metabolites, mainly in somatic organs: neurotoxic intermediate metabolites via main pathway and neuroprotective end product, kynurenic acid (KYNA) via the branch. Elevated levels of KYN have been found in patients with depression. Herein, we investigated whether and how caffeine prevents depression, focusing on the kynurenine pathway. Mice exposed to chronic mild stress (CMS) exhibited depressive-like behaviours with an increase and decrease in plasma levels of pro-neurotoxic KYN and neuroprotective KYNA, respectively. However, caffeine rescued CMS-exposed mice from depressive-like behaviours and restored the plasma levels of KYN and KYNA. Concomitantly, caffeine induced a key enzyme converting KYN into KYNA, namely kynurenine aminotransferase-1 (KAT1), in murine skeletal muscle. Upon caffeine stimulation murine myotubes exhibited KAT1 induction and its upstream PGC-1α sustainment. Furthermore, a proteasome inhibitor, but not translational inhibitor, impeded caffeine sustainment of PGC-1α, suggesting that caffeine induced KAT1 by inhibiting proteasomal degradation of PGC-1α. Thus, caffeine protection against CMS-induced depression may be associated with sustainment of PGC-1α levels and the resultant KAT1 induction in skeletal muscle, and thereby consumption of pro-neurotoxic KYN.

## Introduction

Depression is one of the most common causes of disability worldwide and greatly contributes to the global disease burden. The incidence of depression among adults in high-income countries is approximately 15%, with more than 300 million people affected globally^1,2^. Due to the increasing incidence and considerable social and economic burden associated with this condition, developing strategies for the prevention and treatment of depression represents a public health priority^3,4^; however, over 65% of current antidepressant therapies have been proven ineffective^5,6^.

Increase in kynurenine (KYN), presumably due to the imbalance between main pathway of the kynurenine pathway leading to nicotinamide adenine dinucleotide (NAD^+^) and a particular branch of it producing kynurenic acid (KYNA) (Supplementary Fig. 1), has been reported in patients with brain diseases, including depression^7–11^. The kynurenine pathway serves as a key metabolic process of tryptophan, a diet-derived essential amino acid^12^ that is primarily metabolised in various somatic organs, but only a little in the brain^13^. Indoleamine 2,3-dioxygenase (IDO) and tryptophan 2,3-dioxygenase (TDO), the two initial rate-limiting enzymes in the kynurenine pathway, degrade tryptophan into KYN. KYN is catalysed into two different neurological metabolites by the distinct and independent enzymes, kynurenine 3-monooxygenase (KMO) and kynurenine aminotransferase (KAT). KMO converts KYN into neurotoxic kynurenine metabolites^12,14^, whereas KAT yields KYNA that could be protective or harmful depending on the circumstances^14,15^. As with KYN, some of the neurotoxic kynurenine metabolites, such as 3-hydroxykynurenine, can be transported into the brain across the blood brain barrier (BBB), whereas KYNA cannot^16,17^. Conceivably, KAT-mediated consumption of KYN leads to a reduction in KYN and the resultant neurotoxic KYN metabolites in the brain, as well as in the circulatory system^18^. Indeed, plasma levels of KYNA and the neuroprotective ratio, which is defined as plasma KYNA levels divided by plasma KYN levels, have been reported to be lower in depressed patients than in healthy controls^11^. Thus, activation of KAT in the somatic organs may represent a therapeutic target for preventing depression.

Epidemiological evidence supports the hypothesis that depression is associated with certain dietary factors^19^. Coffee^20^, tea^21^, and caffeine intake have been associated with a decreased risk of depression^20,22^. Caffeine, a bioactive constituent of coffee and tea, has been demonstrated to improve depressive symptoms in humans, as well as in animal models of depression^23^. Adenosine is a neuromodulator that substantially regulates synaptic transmission and neuronal excitability. Adenosine receptors, such as A_1_ and A_2A_ receptors, are expressed in the brain, including in the regions involved in the regulation of cognition, motivation, and emotion^24^. Notably, adenosine receptor-mediated signalling is regarded as being impaired in depression patients^24^. Caffeine is a nonselective A_1_ and A_2A_ adenosine receptor antagonist, which likely participates in the improvement of depression^23^. Indeed, caffeine and other selective adenosine receptor antagonists have been proposed as therapeutic agents for the treatment of motivational dysfunction in depression^23,25^. However, it remains to be elucidated whether and how caffeine modulates the kynurenine pathway to protect against depression.

Recently, exercise has been shown to protect against stress-induced depression by inducing peroxisome proliferator-activated receptor-gamma coactivator 1α (PPARγC1α, hereafter referred to as PGC-1α), a key transcriptional cofactor for KAT, in the skeletal muscle of mice^18^. We previously reported that caffeine supplementation is capable of inducing an exercise-like response in the skeletal muscle of mice^26^. Indeed, exercise activates IL-6 in the skeletal muscle of both mice and humans^27,28^. Intriguingly, mice administered with caffeine in drinking water exhibited selective induction of *Il6* in their skeletal muscle^26^. This feature, common to exercise training and caffeine intake, prompted us to investigate whether caffeine prevents stress-induced depression by activating the PGC-1α-KAT axis in the skeletal muscle of mice.

## Results

### Caffeine protects against CMS-induced depressive-like behaviour in mice

**Figure 1:**
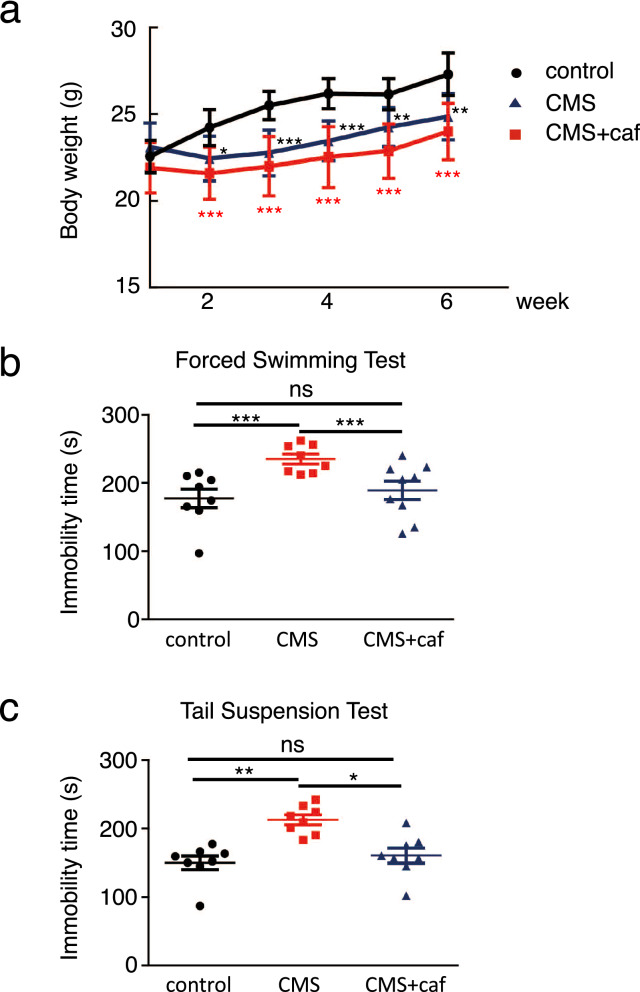
Caffeine protected against CMS-induced depression-like behaviours. Mice were exposed to CMS and were provided with drinking water supplemented with (CMS + caf) or without (CMS) caffeine. Non-CMS-exposed mice were used as controls. During CMS exposure, body weight was monitored weekly (**a**). One week after the last CMS exposure, mice were subjected to the forced swimming test (**b**) and the tail suspension test (**c**). Immobility times were recorded (**b**,**c**). Eight mice were used in control and CMS group, and nine mice were used in CMS + caf group. Data are shown as mean ± SEM. Similar results were obtained in two independent experiments. *p < 0.05; **p < 0.01; ***p < 0.001.

To further substantiate the concept that caffeine is capable of protecting against stress-induced depression, we subjected mice to a CMS protocol with caffeine in their drinking water. Exposure to chronic stress has been reported to induce depressive-like symptoms and impair weight gain in mice during normal growth^29^. Consistent with this, when compared with the unexposed controls, mice exposed to CMS showed a reduction in weight gain (Fig. 1a) along with depressive-like behaviours characterised by prolonged immobility in the forced swimming and tail suspension tests (Fig. 1b,c). Although it did not improve the CMS-induced preclusion of weight gain (Fig. 1a), caffeine supplementation significantly shortened the immobility time of CMS-exposed mice in both tests to levels comparable with those in the control mice (Fig. 1b,c). These results suggest that caffeine ameliorates CMS-induced depressive-like behaviours.

**Figure 2:**
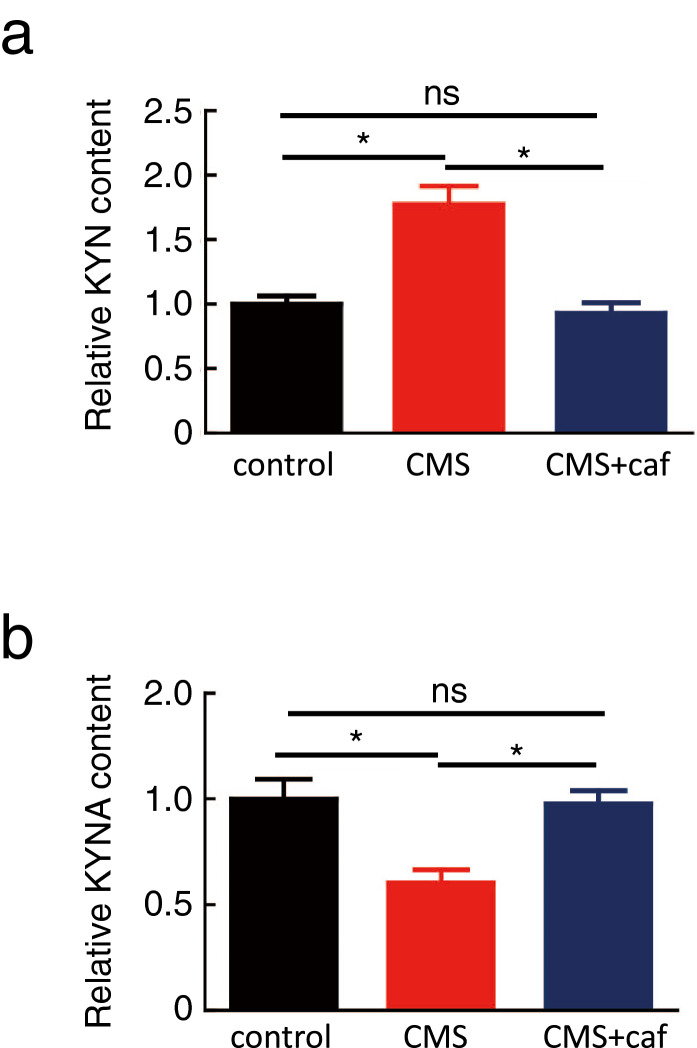
Caffeine restored KYN and KYNA content in plasma. Mice were exposed to CMS with drinking water supplemented with (CMS + caf) or without (CMS) caffeine. Non-CMS-exposed mice were used as controls. After tests for depression-like behaviours, shown in Fig. 1, plasma was sampled for the measurement of KYN and KYNA. Fold changes of KYN (**a**) and KYNA (**b**) to those of control mice were shown. The absolute values of KYN level were 0.28 ± 0.01 μmol/L, 0.49 ± 0.01 μmol/L, and 0.26 ± 0.01 μmol/L for control, CMS, and CMS + caf group, respectively; and the absolute values of KYNA level were 0.32 ± 0.01 μmol/L, 0.19 ± 0.01 μmol/L, and 0.31 ± 0.01 μmol/L for control, CMS, and CMS + caf group, respectively. Eight mice were used in control and CMS group, and nine mice were used in CMS + caf group. Data are shown as mean ± SEM. Similar results were obtained in two independent experiments. *p < 0.05.

Next, we evaluated the circulating levels of KYN and KYNA, since mice that received CMS have been shown to exhibit increased and decreased plasma concentrations of KYN and KYNA, respectively^18^. Consistent with this report, the plasma concentrations of KYN and KYNA in CMS-exposed mice were higher and lower, respectively, than those of non-CMS-exposed control mice (Fig. 2a,b). In contrast, caffeine administration restored plasma KYN and KYNA concentrations to levels comparable with those in the control mice (Fig. 2a,b). These results suggest that caffeine restores the levels of KYN and KYNA.

### Caffeine induces kynurenine aminotransferase expression in skeletal muscle tissue

**Figure 3:**
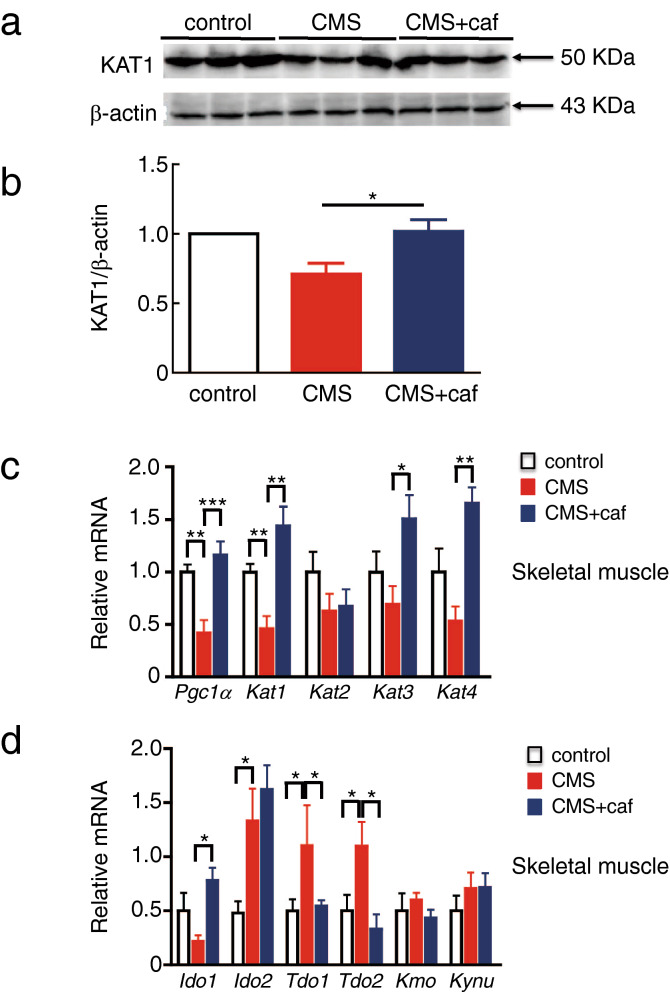
Caffeine administration induced KATs in skeletal muscles of CMS-exposed mice. Skeletal muscles were sampled from the mice in each group, as shown in Fig. 2. KAT1 protein levels in the skeletal muscle were analysed by western blotting (**a**), and their relative levels were normalised to the transcript level of β-actin (**b**). Total RNA was extracted from each muscle sample, followed by RT-qPCR for *Pgc-1α* (**c**), *Kat1* (**c**), *Kat2* (**c**), *Kat3* (**c**), *Kat4* (**c**), *Ido1* (**d**), *Ido2* (**d**), *Tdo1* (**d**), *Tdo2* (**d**), *Kmo* (**d**), and *Kynu* (**d**). Eight mice were used in control and CMS group, and nine mice were used in CMS + caf group. Data are shown as mean ± SEM. Similar results were obtained in two independent experiments. *p < 0.05; **p < 0.01; ***p < 0.001.

KATs catalyse the conversion of KYN to KYNA, resulting in the consumption of KYN along with generation of KYNA^7^. Exercise training leads to higher levels of transcripts and proteins of KATs^18,30,31^, allowing us to investigate whether caffeine stimulates skeletal muscle to express KAT in CMS-exposed mice. There are four KAT isoforms (KAT1 to KAT4) in mice^32^. Western blot analysis revealed that CMS exposure reduced KAT1 levels in the skeletal muscle and that caffeine treatment restored the KAT1 levels in the skeletal muscle (Fig. 3a,b; Supplementary data). In agreement with these findings, RT-qPCR analysis indicated that, compared to unexposed control mice, *Kat1* transcript levels were enhanced by caffeine treatment in CMS-treated animals. Moreover, caffeine supplementation enhanced *Kat3* and *Kat4*, but not *Kat2*, expression levels (Fig. 3c). These results suggest that muscular *Kat* alterations may result in reduced plasma levels of KYNA in CMS-exposed mice and their restoration upon caffeine supplementation (Fig. 2b).

It has been demonstrated that exercise activates skeletal muscle to express PGC-1α, which in turn induces KAT expression^18^. In the present study, we measured *Pgc-1α* transcript levels in the skeletal muscle and, compared to the controls, CMS-exposed mice exhibited a significantly reduced level of *Pgc-1α* (Fig. 3a); however, caffeine administration enhanced *Pgc-1α* levels in the skeletal muscle of CMS-exposed mice (Fig. 3a; Supplementary data). IDO1, IDO2, TDO1, and TDO2 are rate-limiting enzymes in the kynurenine pathway^13,14^. The RT-qPCR results indicated that *Ido2*, *Tdo1*, and *Tdo2* transcript levels were upregulated in the skeletal muscle of CMS-exposed mice compared to those of the control mice (Fig. 3d). However, when CMS-exposed mice were treated with caffeine, the muscular levels of *Tdo1* and *Tdo2* were reduced to levels similar to those of the control mice (Fig. 3d). Intriguingly, mRNA levels of KMO and KYNU, enzymes involved in the other catalytic KYN pathway (Supplementary Fig. 1), were not affected by CMS exposure or caffeine supplementation (Fig. 3d), suggesting that caffeine activated the KAT-mediated pathway for KYNA. Taken together, these results suggest that caffeine administered through drinking water improves the kynurenine pathway and PGC-1α expression levels in murine skeletal muscle.

### Caffeine enhances Kat expression levels in murine myotubes

**Figure 4:**
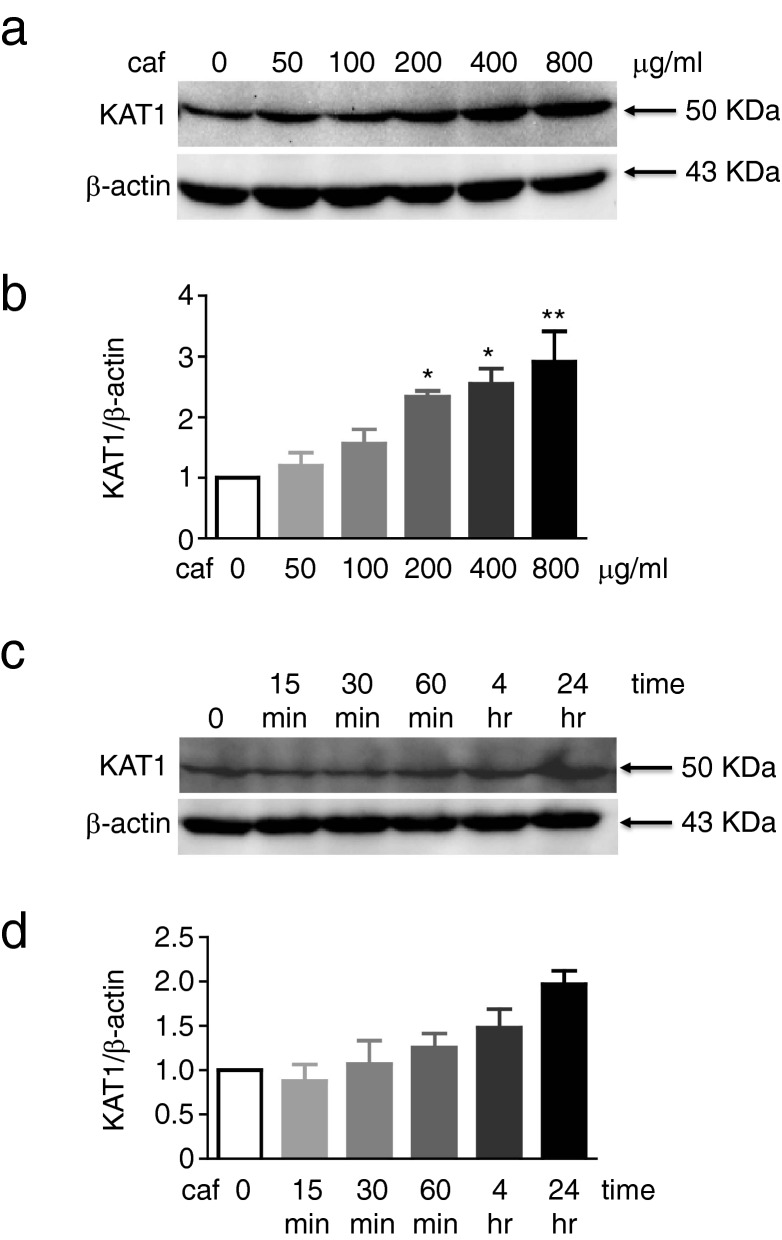
Caffeine induction of both KATs and PGC-1α in myotubes. C2C12 myotubes were incubated with various doses (**a**) or 400 μg/mL (**c**,**d**) of caffeine, for various durations (**c**) or 24 h (**a**,**b**), and cell lysates were prepared for western blotting analysis of KAT1 and β-actin (**a**,**c**). KAT1 images were normalised to the transcript level of the individual β-actin, and the relative ratio to that of the control (0 min) was calculated (**b**,**d**). Data are shown as mean ± SEM of the triplicate experiments. Similar results were obtained in three independent experiments. *p < 0.05; **p < 0.01.

**Figure 5:**
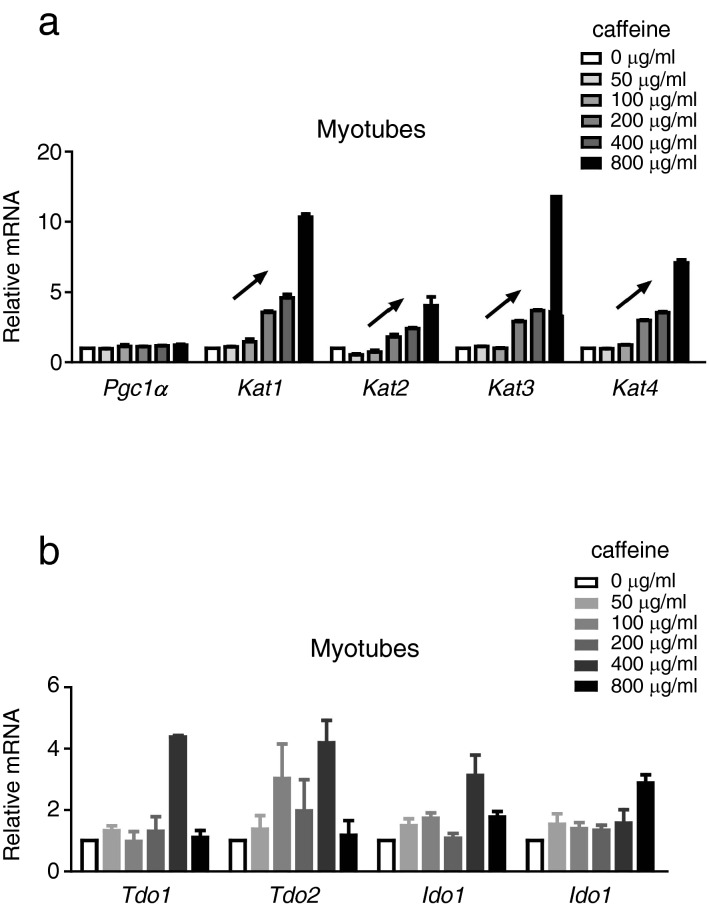
Caffeine induction of Kats but not Pgc-1α in myotubes. C2C12 myotubes were incubated with 400 μg/mL of caffeine for 24 h, and total RNA was prepared, followed by RT-qPCR for *Pgc-1α* (**a**), *Kat1* (**a**), *Kat2* (**a**), *Kat3* (**a**), *Kat4* (**a**), *Tdo1* (**b**), *Tdo2* (**b**), *Ido1* (**b**), and *Ido2* (**b**). Each gene was normalised to the transcript level of β-actin, and relative mRNA to control (0 mg/mL) was calculated (**a**,**b**). Data are shown as mean ± SEM of the triplicate experiments. Similar results were obtained in three independent experiments. *p < 0.05; **p < 0.01; ***p < 0.001.

We wanted to know whether caffeine directly induces *Kats* in skeletal muscle. To test this, we incubated murine myotubes with various doses of caffeine for 24 h, or 400 μg/mL of caffeine for different durations. Western blotting revealed that caffeine treatment induced KAT1 levels in a dose- and duration-dependent manner (Fig. 4a–d; Supplementary data). Moreover, caffeine activated murine myotubes to enhance *Kat1*, *Kat2*, *Kat3*, and *Kat4* (Fig. 5a), but not *Ido1*, *Ido2*, *Tdo1*, or *Tdo2* (Fig. 5b). These results suggest that caffeine directly induces *Kats*, but not *Idos* or *Tdos*, in murine skeletal muscle.

### Caffeine increases PGC-1α levels by inhibiting proteasomal degradation

**Figure 6:**
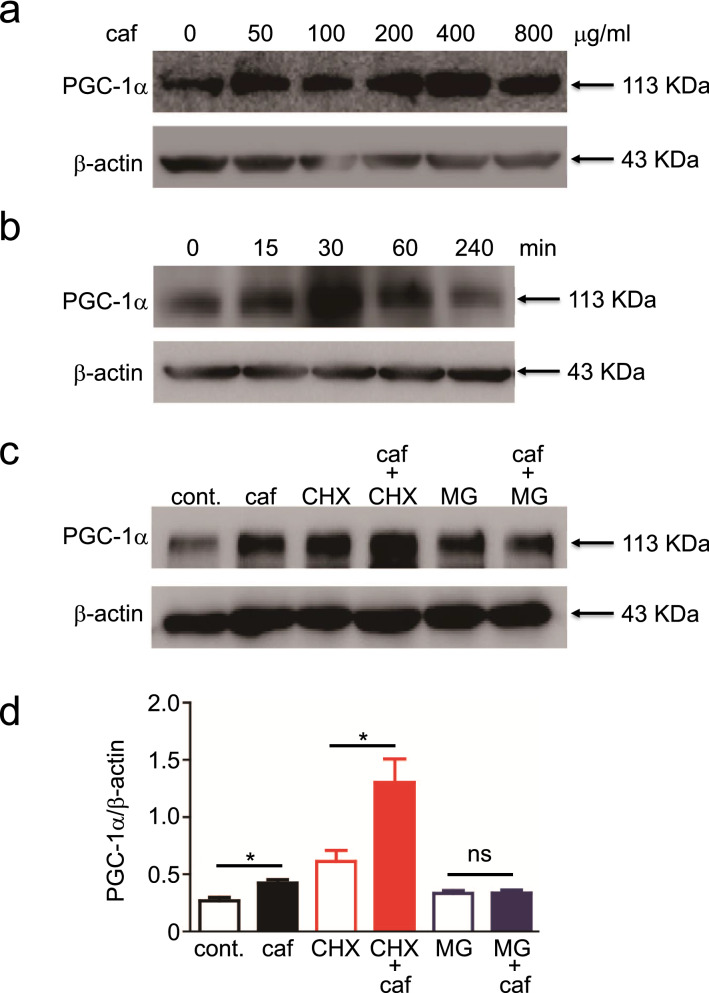
Caffeine enhanced PGC-1α by inhibiting its proteasomal degradation. (**a**,**b**) C2C12 myotubes were incubated with various doses (**a**) or 400 μg/mL (**b**) of caffeine for various durations (**b**) or 24 h (**a**). (**c**,**d**) The myotubes were preincubated with cycloheximide (CHX) or MG132 (MG) for 1 h, followed by incubation with 400 μg/mL caffeine (caf) for 24 h. PGC-1α and β-actin levels were analysed by western blotting (**a**–**c**). PGC-1α images (**c**) were normalised to the transcript level of the individual β-actin (**d**). Data are shown as mean ± SEM of the triplicate experiments. Similar results were obtained in three independent experiments. *p < 0.05.

**Figure 7:**
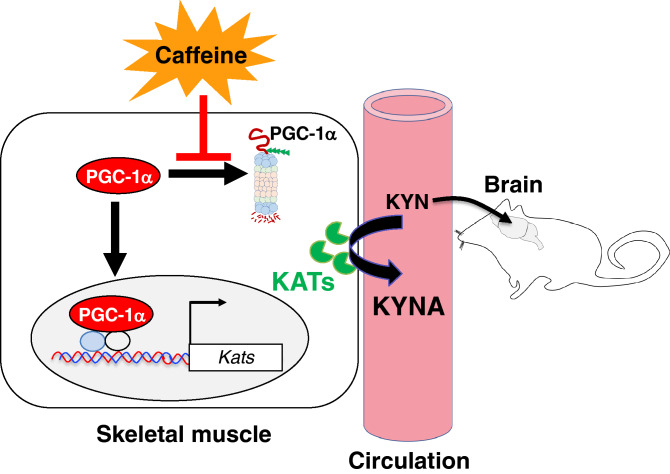
Proposed model for the protective role of caffeine against CMS-induced depression. CMS-exposed mice showed depressive-like behaviour with an increase in plasma levels of kynurenine (KYN). Caffeine supplementation protected against depressive behaviours in CMS-exposed mice, accompanied by restoration of plasma concentrations of BBB-permeable, pro-neurotoxic KYN, and BBB-impermeable KYNA. In addition, caffeine administration activated kynurenine aminotransferase (KAT) which consumed KYN by converting it to KYNA in the skeletal muscles of the mice. Indeed, caffeine stimulated myotubes to sustain PGC-1α, a transcription factor for KAT, by inhibiting their proteasomal degradation. These events converge on KAT-mediated consumption of KYN, eventually leading to a reduction in plasma and brain levels of KYN.

Because PGC-1α has been reported to induce KAT expression^31^, we investigated whether caffeine treatment can enhance PGC-1α levels in murine myotubes. To validate the anti-PGC-1α antibody specificity, we generated *Pgc1α*^−/−^ C2C12 cells using CRISPR/Cas9 technology. We performed western blotting using lysates prepared from parental *Pgc1α*^+/+^ and *Pgc1α*^−/−^ C2C12 cells and found that the band for PGC-1α was visible on PGC1α-sufficient but not on *Pgc1α*^−/−^ samples (Supplementary Fig. 2), verifying the specificity of the antibody used. We found that caffeine treatment upregulated PGC-1α protein levels in a dose-dependent manner (Fig. 6a; Supplementary data). Kinetic analysis revealed that the level of PGC-1α peaked at 15 to 30 min, followed by a rapid reduction to the base level by 4 h post-incubation with caffeine (Fig. 6b; Supplementary data). Interestingly, cycloheximide, an inhibitor of translation, did not dampen the caffeine-induced increase in the PGC-1α level (Fig. 6c,d; Supplementary data), strongly indicating a low requirement of de novo protein synthesis for caffeine-induced PGC-1α. Consistent with this, caffeine exposure did not induce *Pgc-1α* in the murine myotubes (Fig. 5a). In contrast, MG132, a proteasome inhibitor, suppressed the caffeine induction of PGC-1α (Fig. 6d). Taken together, these results suggest that caffeine upregulates PGC-1α levels by inhibiting proteasomal degradation, which then evokes KAT1 in the skeletal muscle, eventually leading to the reduction in circular KYN and presumably brain KYN through the conversion of BBB-permeable KYN to BBB-impermeable KYNA (Fig. 7).

## Discussion

Consistent with previously reported protective effects of physical exercise^18^, we found that caffeine treatment prevented depression-like behaviours in CMS-exposed mice (Fig. 1b,c). Caffeine intake promoted the conversion of KYN to KYNA (Figs. 2a,b, 7), presumably by inducing KAT1 expression in the skeletal muscle (Figs. 3, 7), and activated myotubes to express robust PGC-1α (Figs. 6, 7). Our results suggest that caffeine may act on skeletal muscle and activate the PGC-1α–KAT axis; this would promote the consumption of BBB-permeable KYN, thereby preventing damage to the brain. In addition to its modulation of adenosine and dopamine receptors in the brain^23^, caffeine restoration of the kynurenine pathway in skeletal muscle may participate in protection against depression.

Patients with major depression are often associated with body weight loss^33^, as was observed in our CMS-treated mice (Fig. 1a). Antidepressant use is linked with increased obesity, and many psychotropic drugs can affect appetite, eating behaviour, and body weight^34^; however, caffeine treatment did not rescue body weight loss in CMS-exposed mice. Caffeine is known to increase energy expenditure and decrease energy intake, and can be used to better maintain body weight (reviewed in^35^). These observations may explain why caffeine intake failed to rescue body weight loss in our CMS-treated mice, further supporting the idea that, compared to other antidepressants, caffeine can improve motivational dysfunction in depression without causing excessive body weight gain.

PGC-1α is an inducible transcriptional coactivator that regulates mitochondrial biogenic and cellular energy metabolic pathways^36^. Loss of body weight in caffeine-treated mice may result from PGC-1α activation, although the mechanism through which caffeine induces PGC-1α expression remains unclear. With a half-life of 0.3 h, PGC-1α is rapidly degraded in the nucleus via the ubiquitin proteasome system^37^, likely explaining why caffeine induced PGC-1α levels within the 15–30 min timeframe. Cytokines^38^, acetylation^39^, and the ubiquitin proteasome pathway are all reportedly involved in PGC-1α degradation^40^, while the p38 MAPK and AMPK pathways have also been implicated in the control of PGC-1α expression in skeletal muscle after exercise training^41,42^. Therefore, further investigation is needed to clarify how caffeine regulates the proteasomal degradation of PGC-1α.

TDO1 and TDO2 are key and leading enzymes of the kynurenine pathway, and were substantially upregulated in CMS-exposed animals (Fig. 3d). Glucocorticoid induction has been reported to increase TDOs but not IDOs^43^. CMS-exposed mice have been reported to have increased plasma levels of corticosterone^44^. Furthermore, CMS-exposed mice were previously found to have lost corticosterone circadian oscillation, which is finely tuned by the hypothalamic–pituitary–adrenal axis^44,45^. It is also worth noting that adenosine A_2A_ receptor antagonists can reverse the hypothalamic–pituitary–adrenal axis and restore corticosterone circadian rhythm^46^. We found that caffeine supplementation restored TDOs in the skeletal muscles of CMS-exposed mice (Fig. 3d), which may be attributed to caffeine reduction in circulating corticosterone levels.

CMS-exposed mice showed reduced *Ido1* expression in their skeletal muscle (Fig. 3d). However, caffeine supplementation restored *Ido1* expression in the skeletal muscle of CMS-exposed mice (Fig. 3d). It is well established that proinflammatory cytokines are capable of inducing IDO1^47^. In particular, IL-6 has been reported to be capable of inducing *Ido1* in a manner dependent on its transcription factor STAT3^48,49^. As previously reported, mice administered with caffeine in drinking water can induce *IL6* in their skeletal muscles, which eventually protects against diet-induced non-alcoholic fatty liver diseases^26^. In addition, murine myotubes secrete IL-6 upon stimulation with caffeine in a manner dependent on p38 MAPK^26^. Thus, the restoration of *Ido1* in the skeletal muscles of caffeinated CMS-exposed mice may be a signature of caffeine induction of muscular IL-6.

This study revealed that caffeine protected against CMS-induced depression possibly through preferential activation of the branch of the kynurenine pathway, which is the conversion to KYNA, in the skeletal muscle. However, this study contains several limitations. First, although caffeine supplementation increased plasma levels of KYNA with induction of KATs in the skeletal muscle, the systemic organs other than skeletal muscles, such as liver, might participate in this response via activation of KAT or other enzymes as well^13^. Second, brain levels of KYN and KMO-mediated metabolites are determined not only by their circulating levels due to their ability to cross the BBB but also the brain-born ones. Caffeine might directly activate the production of these metabolites in the brain. To accurately evaluate the effect of caffeine, we need to measure the amounts of these metabolites in the brain. Third, this study showed that caffeine supplementation did not affect *Kmo* levels in the skeletal muscle. This does not exclude the possibility that caffeine affects the generation of KMO-mediated metabolites. We need further efforts to have broken these limitations in the near future. Beyond these limitations, however, this study might still provide a milestone to generate the novel concept that caffeine protection against CMS-induced depression may be mediated by the activation of PGC-1α and KAT in the skeletal muscle.

The present study demonstrated caffeine as a potent modulator of the kynurenine pathway in skeletal muscle. Caffeine prevented stress-induced depression by prompting the skeletal muscle to exert exercise-like action; namely, caffeine supplementation upregulated KAT1, presumably via inhibition of PGC-1α degradation, which, in turn, restored the kynurenine pathway, eventually leading to the prevention of CMS-exposed depression.

## Methods

### Mice

Male C57BL/6J mice (6–7 weeks old; Cavens Laboratory Animals, Nanjing, China) were housed in a controlled environment (humidity = 50–60%; ambient temperature = 24 ± 1 °C; 12 h light–dark cycle) and allowed to adapt to their environment for 1 week. Body weight was recorded weekly. All experimental procedures were carried out in accordance with the guidelines set by the Committee for the Care and Use of Laboratory Animals of Yunnan Agricultural University (Kunming, China)^50^. All procedures were approved by the Animal Experiments Ethics Committee of Yunnan Agricultural University^50^. The study was carried out in compliance with the ARRIVE guidelines (http://www.nc3rs.org.uk/page.asp?id=1357).

### Induction of chronic mild stress-induced depression

Mice were subjected to chronic mild stress (CMS)^18^, either without or with caffeine at a level of 0.5 mg/mL in their drinking water, for 6 weeks. The CMS protocol is shown in Supplementary Table 1. Mice were subjected to depression-like behaviour tests (described below). After the tests, the mice were euthanized, followed by collection of their plasma and skeletal muscles.

### Forced swim test

Mice were placed in a cylinder containing 20 cm of water and allowed to adapt to the swimming environment for 15 min. After 24 h, the mice were again placed in the cylinder and immobility time (including movements to stay afloat) during a 5 min period was recorded.

### Tail suspension test

Mice were suspended by the tail with adhesive tape placed 2 cm from the tip, with the nose of the mouse being 5 cm from the floor. The immobility time during a 5 min period was recorded.

### Cell culture

As shown previously^26^, the mouse C2C12 myoblast cell line was obtained from American Type Culture Collection (ATCC; Manassas, VA, USA) and cultured in Dulbecco's modified Eagle's medium (DMEM) supplemented with 10% foetal bovine serum^26^. For differentiation of C2C12 myotubes, cells were cultured in DMEM containing 2% horse serum for 7 days. Cells were cultured at 37 °C in a humidified atmosphere with 5% CO_2_.

### Generation of *Pgc1α*^−/−^ C2C12 cells using CRISPR/Cas9 technology

We generated *Pgc1α*^−/−^ C2C12 cells according the method described elsewhere^26^. A PGC-1α-knockout cell line derived from C2C12 cells was constructed using the CRISPR/Cas9 system. Expression plasmids containing a Cas9 nickase and paired gRNA targeting mouse PGC-1α (5′-GTAGCTGAGCTGAGTGTTGGC-3′) was used according to the manufacturer’s instructions (Hanbio Biotechnology, Shanghai, China). C2C12 cells were transfected with the expression plasmids using the Amaxa Nucleofector System (Lonza, Allendale, NJ, USA) according to the manufacturer’s instructions, and cultured. Single colonies were then selected and passaged, and target sequences were analyzed using a 3100 Genetic Analyzer system (Applied Biosystems). PGC-1α protein expression levels in each clone were examined using western blotting as described above (Supplementary Fig. 2). We obtained one *Pgc1α*^−/−^ clone (Supplementary Fig. 2).

### Western blotting

We performed western blotting analysis according to the methods shown elsewhere^50^. At the end of the animal experiment, mice were sacrificed by isoflurane inhalation, and the skeletal muscle and plasma were sampled and stored at − 80 °C. Cell lysates were prepared from cultured C2C12 myotubes or skeletal muscle tissue using RIPA buffer (Solarbio, Beijing, China). In some experiments, we generated cell lysates of *Pgc1α*^+/+^ parental C2C12 cells and *Pgc1α*^−/−^ C2C12 clone cells. Protein concentration was determined using a bicinchoninic acid assay. Proteins were separated using SDS-PAGE and then transferred to a polyvinylidene fluoride membrane (Millipore, Bedford, MA, USA). The membrane was washed, blocked, and subjected to primary antibody incubation before being incubated with a corresponding horseradish peroxidase-conjugated secondary antibody (Santa Cruz, Dallas, USA). In some experiments, the membranes were cut before hybridization with antibodies to shorten the blot process (i.e. Fig. 3a β-actin, Fig. 6c PGC-1α, and figure S2 β-tubulin), so the full-length blots were not provided.

Protein bands were detected using a Pro-light Horseradish Peroxidase Chemiluminescence kit (Tiangen Biotech, Beijing, China), according to the manufacturer's instructions. Images were acquired using a FluorChem E system (ProteinSimple, Santa Clara, CA, USA). The anti-PGC-1α antibody was purchased from Millipore (Cat. No. ST-1202), while the anti-KAT1 antibody was obtained from Proteintech (Chicago, IL, USA, Cat. No. 12156-1-AP)**.**

### Quantitative reverse transcription PCR (RT-qPCR)

We performed RT-qPCR as described elsewhere^50^. Total RNA was extracted from skeletal muscle tissue and C2C12 myotubes using TransZol Up (TransGen Biotech, Beijing, China), following the manufacturer’s instructions. Total RNA was reverse transcribed using a PrimeScript RT Reagent kit with gDNA Eraser (TaKaRa Biotechnology, Kusatsu, Japan), according to the manufacturer’s instructions. Quantitative RT-qPCR was performed using a SYBR Premix Ex Taq II kit (Tli RNase H Plus; TaKaRa Biotechnology), and the results were determined using a 7900HT Fast Real-Time PCR system (Applied Biosystems, Foster City, CA, USA). Data were calculated using the comparative 2^−ΔΔCt^ method, and all values were normalised to the transcript level of the endogenous β-actin gene. The PCR mixture consisted of 0.3 mM primers, cDNA, ROX Reference, and SYBR green mix (Platinum SYBR Green qPCR SuperMix-UDG; Invitrogen, Raritan, New Jersey, United States), with a total reaction volume of 10 μL. Primer sequences (Generay Biotech, Shanghai, China) are provided in Supplementary Table 2.

### High-performance liquid chromatography

Trifluoroacetic acid (TFA) was used for protein precipitation in plasma sample preparation. Plasma levels of KYN and KYNA were assessed using a 1260 high-performance liquid chromatography (HPLC) system (Agilent, Palo Alto, CA, USA) equipped with a quaternionic pump, autosampler, UV (365 nm) detector, and fluorescence detector (excitation 344 nm; emission 398 nm). A Zorbax SB-C18 column (250 mm × 4.6 mm i.d., 5 μm) was used for separation. The mobile phase consisted of a mixture of 10 mM acetate buffer and acetonitrile (93:7). Flow rate was 1 mL/min.

### Statistical analysis

We performed the statistical analysis described previously^26^. All data are presented as mean ± SEM. Differences between groups were evaluated using one-way ANOVA followed by Tukey’s test. A *P*-value of < 0.05 was considered significant. Each experiment was repeated independently at least three times, with similar results. Representative data are shown.

## Supplementary Information

Supplementary Information.

## References

[1] Bromet E (2011). Cross-national epidemiology of DSM-IV major depressive episode. BMC Med..

[2] Lim GY (2018). Prevalence of depression in the community from 30 countries between 1994 and 2014. Sci. Rep..

[3] Fava M (2000). Management of nonresponse and intolerance: Switching strategies. J. Clin. Psychiatry.

[4] John Rush A (2006). Bupropion-SR, sertraline, or venlafaxine-XR after failure of SSRIs for depression. N. Engl. J. Med..

[5] Crown WH (2002). The impact of treatment-resistant depression on health care utilization and costs. J. Clin. Psychiatry.

[6] Fava M (2003). Diagnosis and definition of treatment-resistant depression. Biol. Psychiatry.

[7] Oxenkrug GF (2010). Tryptophan–kynurenine metabolism as a common mediator of genetic and environmental impacts in major depressive disorder: The serotonin hypothesis revisited 40 years later. Isr. J. Psychiatry Relat. Sci..

[8] Claes S (2011). The kyurenine pathway in major depression: Haplotype analysis of three related functional candidate genes. Psychiatry Res..

[9] Smith AK (2012). Association of a polymorphism in the indoleamine-2,3-dioxygenase gene and interferon-alpha-indused depression in patients with chronic hepatitis C. Mol. Psychiatry.

[10] Yu C-P, Pan Z-Z, Luo D-Y (2016). TDO as a therapeutic target in brain diseases. Metab. Brain Dis..

[11] Myint A-M (2007). Kynurenine pathway in major depression: Evidence of impaired neuroprotection. J. Affect. Disord..

[12] Platten M, Nollen EAA, Röhrig UF, Fallarino F, Opitz CA (2019). Tryptophan metabolism as a common therapeutic target in cancer, neurodegeneration and beyond. Nat. Rev. Drug Discov..

[13] Cervenka I, Agudelo LZ, Ruas JL (2017). Kynurenines: Tryptophan's metabolites in exercise, inflammation, and mental health. Science.

[14] Schwarcz R, Bruno JP, Muchowski PJ, Wu H-Q (2012). Kynurenines in the mammalian brain: When physiology meets pathology. Nat. Rev. Neurosci..

[15] Duda W (2019). Interaction of the immune-inflammatory and the kynurenine pathways in rats resistant to antidepressant treatment in model of depression. Int. Immunopharmacol..

[16] Boado RJ, Yi Li J, Nagaya M, Zhang C, Pardridge WM (1999). Selective expression of the large neutral amino acid transporter at the blood–brain barrier. Proc. Natl. Acad. Sci. U.S.A..

[17] Fukui S, Schwarcz R, Rapoport SI, Takada Y, Smith QR (1991). Blood–brain barrier transport of kynurenines: Implications for brain synthesis and metabolism. J. Neurochem..

[18] Agudelo LZ (2014). Skeletal muscle PGC-1alpha1 modulates kynurenine metabolism and mediates resilience to stress-induced depression. Cell.

[19] Murakami K, Sasaki S (2010). Dietary intake and depressive symptoms: A systematic review of observational studies. Mol. Nutr. Food Res..

[20] Wang L, Shen X, Wu Y, Zhang D (2016). Coffee and caffeine consumption and depression: A meta-analysis of observational studies. Aust. NZ J. Psychiatry.

[21] Dong X (2015). Tea consumption and the risk of depression: A meta-analysis of observational studies. Aust. NZ J. Psychiatry.

[22] Grosso G, Micek A, Castellano S, Pajak A, Galvano F (2016). Coffee, tea, caffeine and risk of depression: A systematic review and dose-response meta analysis of observational studies. Mol. Nutr. Food Res..

[23] López-Cruz L, Salamone JD, Correa M (2018). Caffeine and selective adenosine receptor antagonists as new therapeutic tools for the motivational symptoms of depression. Front. Pharmacol..

[24] van Calker D, Biber K, Domschke K, Serchov T (2019). The role of adenosine receptors in mood and anxiety disorders. J. Neurochem..

[25] Fredholm BB, Bättig K, Holmén J, Nehlig A, Zvartau EE (1999). Actions of caffeine in the brain with special reference to factors that contribute to its widespread use. Pharmacol. Rev..

[26] Fang C (2019). Caffeine-stimulated muscle IL-6 mediates alleviation of non-alcoholic fatty liver disease. Biochim. Biophys. Acta Mol. Cell Biol. Lipids.

[27] Pedersen BK, Febbraio MA (2008). Muscle as an endocrine organ: Focus on muscle-derived interleukin-6. Physiol. Rev..

[28] Lightfoot AP, Cooper RG (2016). The role of myokines in muscle health and disease. Curr. Opin. Rheumatol..

[29] Li Z-Q, Yan Z-Y, Lan F-J, Dong Y-Q, Xiong Y (2018). Suppression of NLRP3 inflammasome attenuates stress-induced depression-like behavior in NLGN3-deficient mice. Biochem. Biophys. Res. Commun..

[30] Lewis GD (2010). Metabolic signatures of exercise in human plasma. Sci. Transl. Med..

[31] Pedersen BK (2019). Physical activity and muscle–brain crosstalk. Nat. Rev. Endocrinol..

[32] Agudelo LZ (2019). Skeletal muscle PGC-1alpha1 reroutes kynurenine metabolism to increase energy efficiency and fatigue-resistance. Nat. Commun..

[33] Fernstrom MH (1989). Depression, antidepressants, and body weight change. Ann. N.Y. Acad. Sci..

[34] McElroy SL, Guerdjikova AI, Mori N, Keck PE (2016). Managing comorbid obesity and depression through clinical pharmacotherapies. Expert Opin. Pharmacother..

[35] Harpaz E, Tamir S, Weinstein A, Weinstein Y (2017). The effect of caffeine on energy balance. J. Basic Clin. Physiol. Pharmacol..

[36] Finck BN, Kelly DP (2006). PGC-1 coactivators: Inducible regulators of energy metabolism in health and disease. J. Clin. Investig..

[37] Trausch-Azar J, Leone TC, Kelly DP, Schwartz AL (2010). Ubiquitin Proteasome-dependent degradation of the transcriptional coactivator PGC-1alpha via the N-terminal pathway. J. Biol. Chem..

[38] Puigserver P (2001). Cytokine stimulation of energy expenditure through p38 MAP kinase activation of PPARgamma coactivator-1. Mol. Cell.

[39] Dominy JE, Lee Y, Gerhart-Hines Z, Puigserver P (2010). Nutrient-dependent regulation of PGC-1alpha's acetylation state and metabolic function through the enzymatic activities of Sirt1/GCN5. Biochim. Biophys. Acta.

[40] Sano M (2007). Intramolecular control of protein stability, subnuclear compartmentalization, and coactivator function of peroxisome proliferator-activated receptor gamma coactivator 1alpha. J. Biol. Chem..

[41] Zong H (2002). AMP kinase is required for mitochondrial biogenesis in skeletal muscle in response to chronic energy deprivation. Proc. Natl. Acad. Sci. U.S.A..

[42] Akimoto T (2005). Exercise stimulates Pgc-1alpha transcription in skeletal muscle through activation of the p38 MAPK pathway. J. Biol. Chem..

[43] Badawy AA-B (2017). Kynurenine pathway of tryptophan metabolism: Regulatory and functional aspects. Int. J. Tryptophan Res..

[44] Takahashi K (2013). Chronic mild stress alters circadian expessions of molecular clock genes in the liver. Am. J. Physiol. Endocrinol. Metab..

[45] Menke A (2019). Is the HPA axis as target for depression outdated, or is there a new hope?. Front. Psychiatry.

[46] Batalha VL (2013). Adenosine A2A receptor blockade reverts hippocampal stress-induced deficits and restores corticosterone circadian oscillation. Mol. Psychiatry.

[47] Baumgartner R, Forteza MJ, Ketelhuth DFJ (2019). The interplay between cytokines and the Kynurenine pathway in inflammation and atherosclerosis. Cytokine.

[48] Litzenburger UM (2014). Constitutive IDO expression in human cancer is sustained by an autocrine signaling loop involving IL-6, STAT3 and the AHR. Oncotarget.

[49] Yu J (2014). Noncanonical NF-kappaB activation mediates STAT3-stimulated IDO upregulation in myeloid-derived suppressor cellls in breast cancer. J. Immunol..

[50] Du X (2018). Caffeine promotes conversion of palmitic acid to palmitoleic acid by inducing expression of fat-5 in caenorhabditis elegans and scd1 in mice. Front. Pharmacol..

