# Supplementary material for: Caffeine protects against stress-induced murine depression through activation of PPARγC1α-mediated restoration of the kynurenine pathway in the skeletal muscle
Source: Sci Rep. 2021 Mar 31;11:7287. doi: 10.1038/s41598-021-86659-4 (PMC8012704; doi:10.1038/s41598-021-86659-4)
Supplement: Supplementary file 1 — Supplementary Information. [file 41598_2021_86659_MOESM1_ESM.pdf]

Caffeine protects against stress-induced murine depression through activation of PPARγC1α-mediated restoration of the kynurenine pathway in the skeletal muscle

Chongye Fang, Shuhei Hayashi, Xiaocui Du, Xianbin Cai, Bin Deng, Hongmei Zheng, Satoshi Ishido, Hiroko Tsutsui, Jun Sheng

Supplementary Data

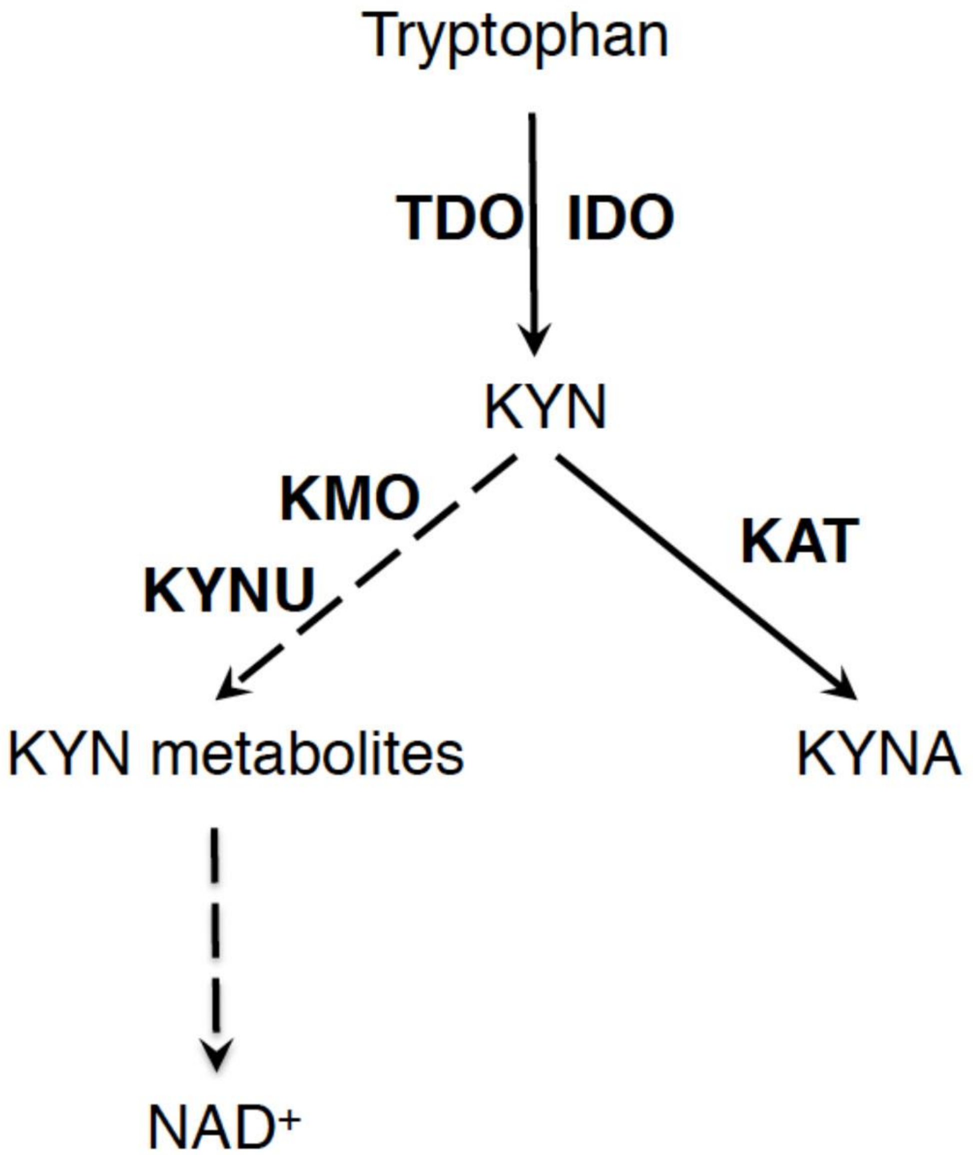

Supplementary Fig. 1: Overview of the Kynurenine pathway

TDD, tryptophan 2,3-dioxygenase; IDO, indoleamine 2,3-dioxygenase; KYN, kynurenine; KMO, kynurenine 3-monooxygenase; KYNU, kynureninase; NAD+, nicotinamide adenine dinucleotide; KAY, kynurenine aminotransferase; KYNA, kynurenic acid.

Full unedited gel for Figure 3a

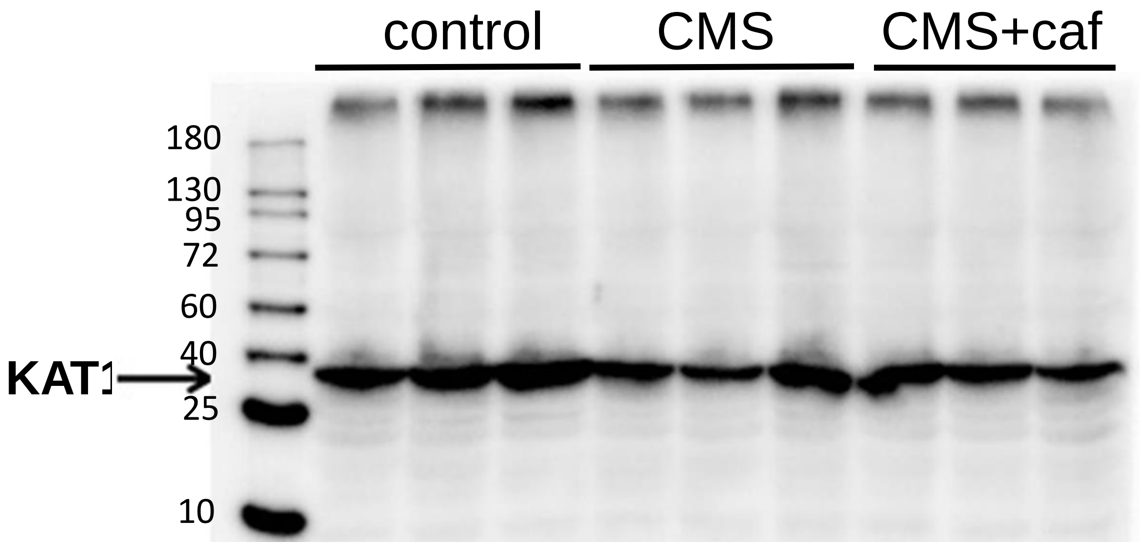

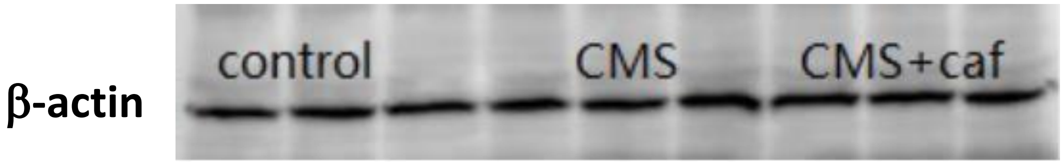

Full unedited gel for Figure 4a

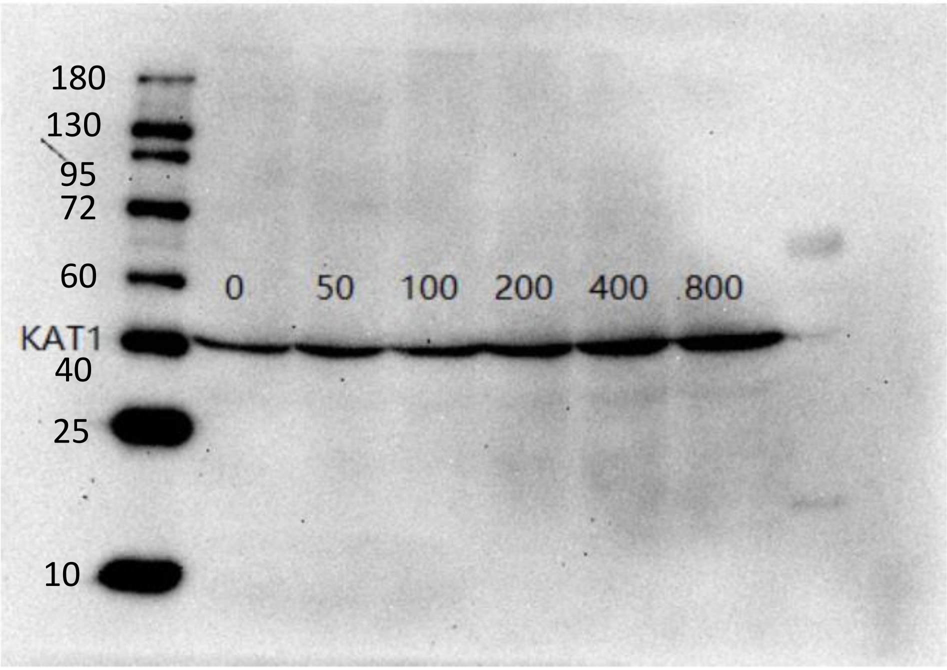

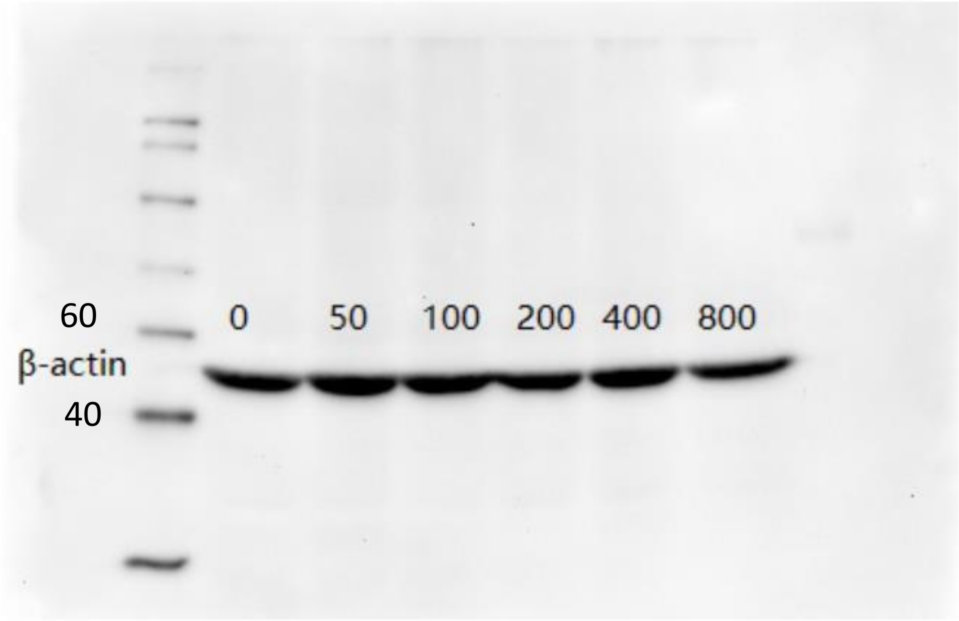

Full unedited gel for Figure 4c

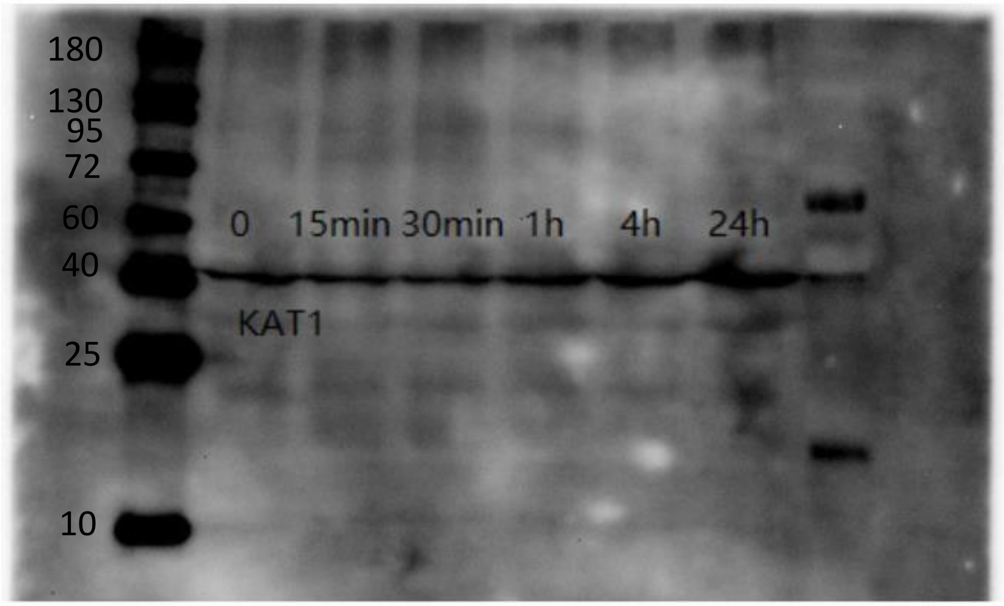

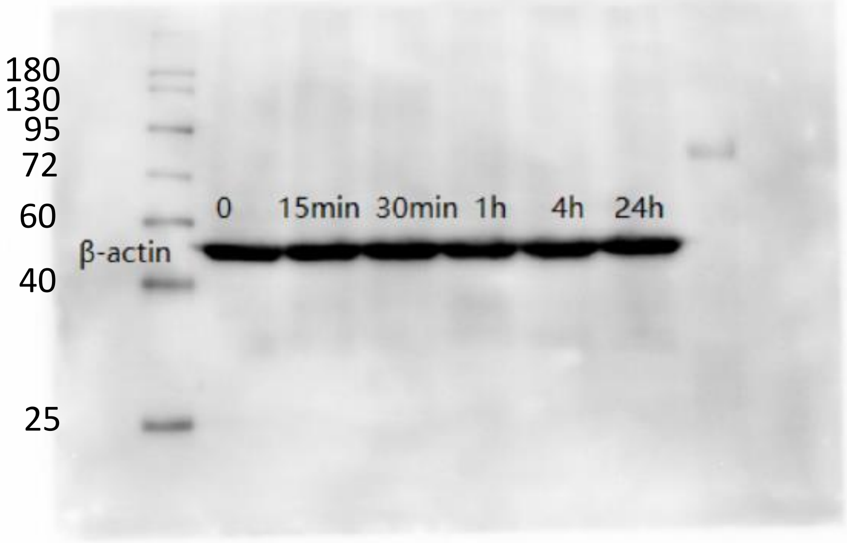

Full unedited gel for Figure 6a

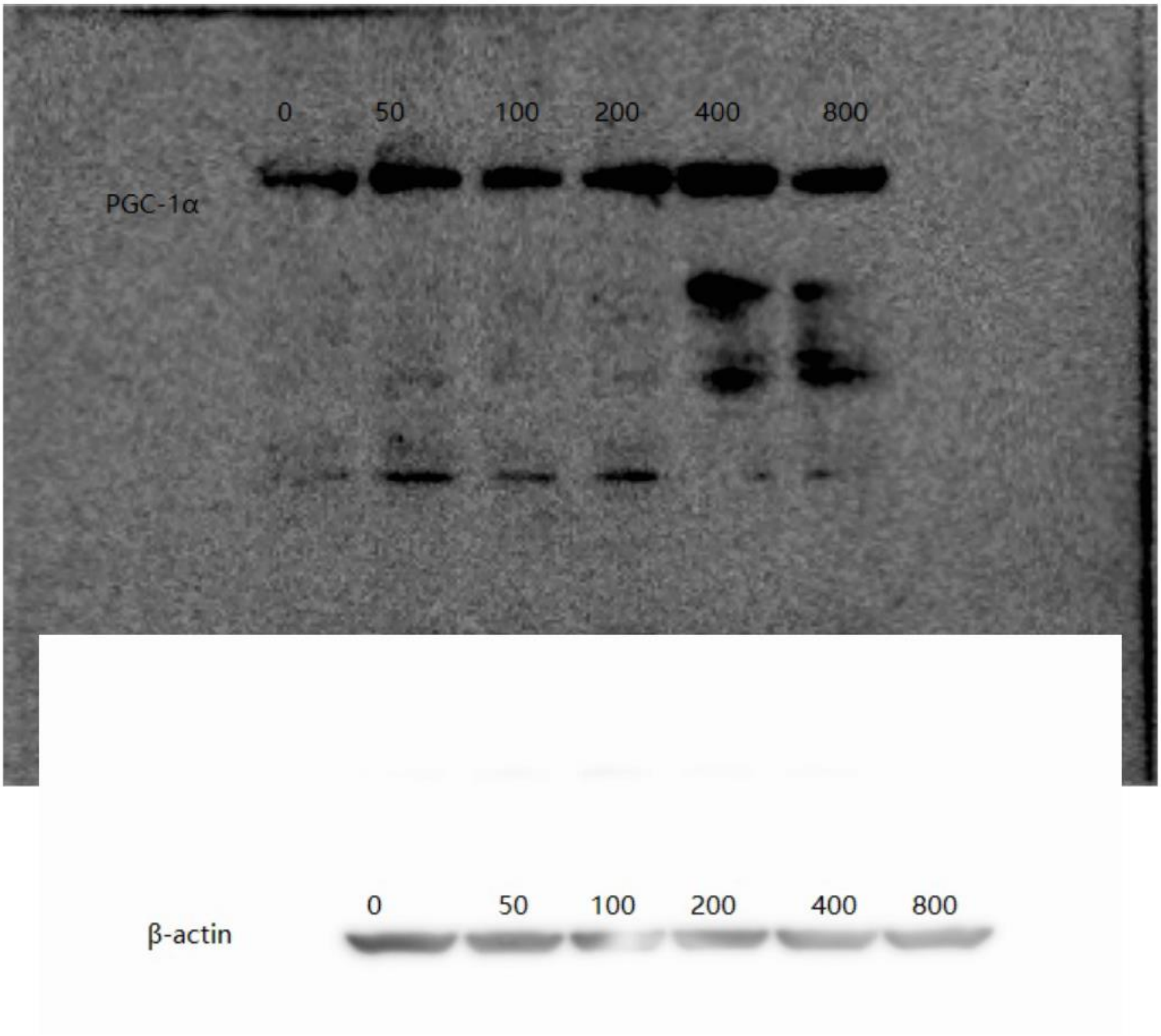

Multiple exposure images for actin of Fig.6a

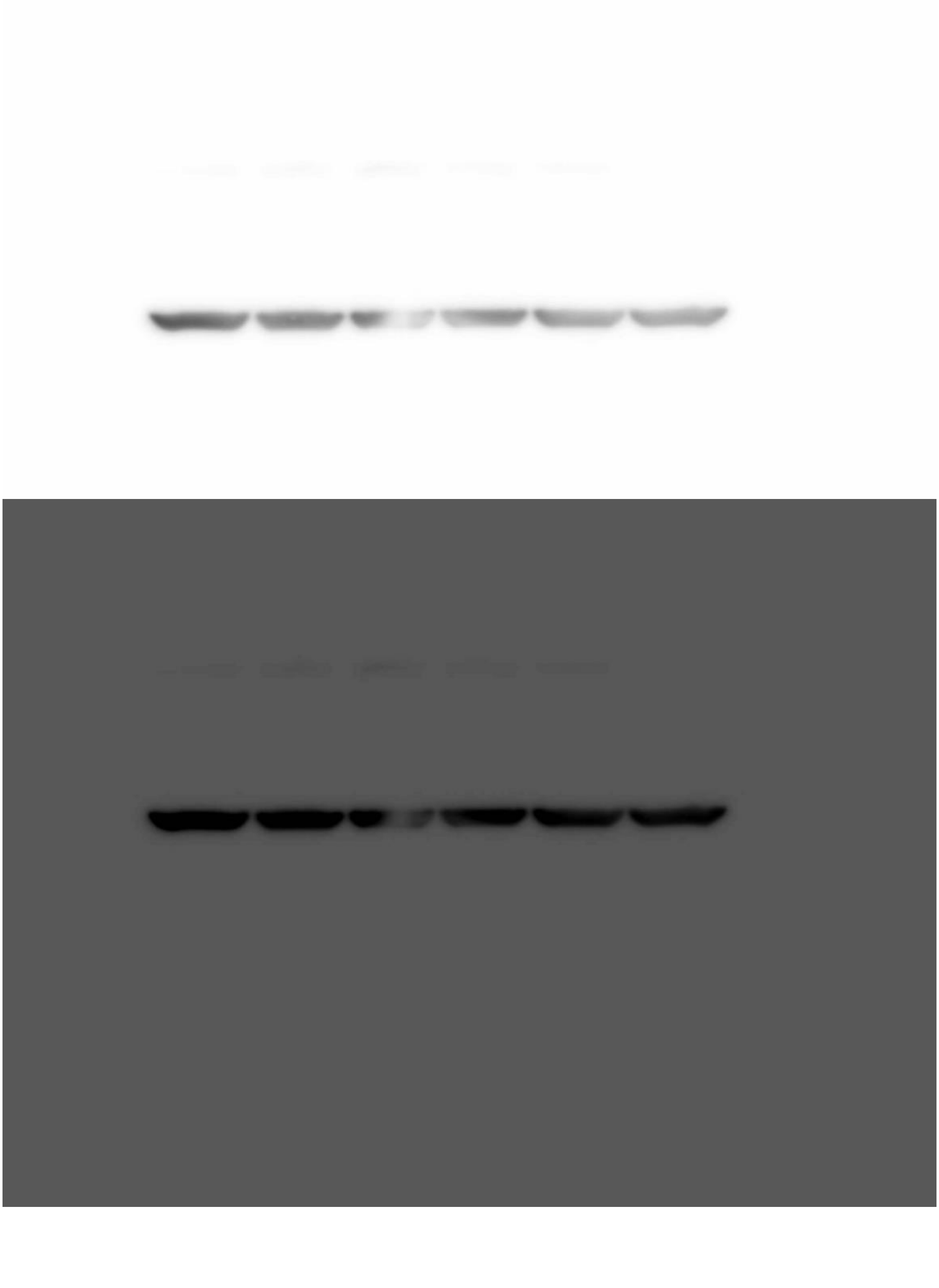

Full unedited gel for Figure 6b

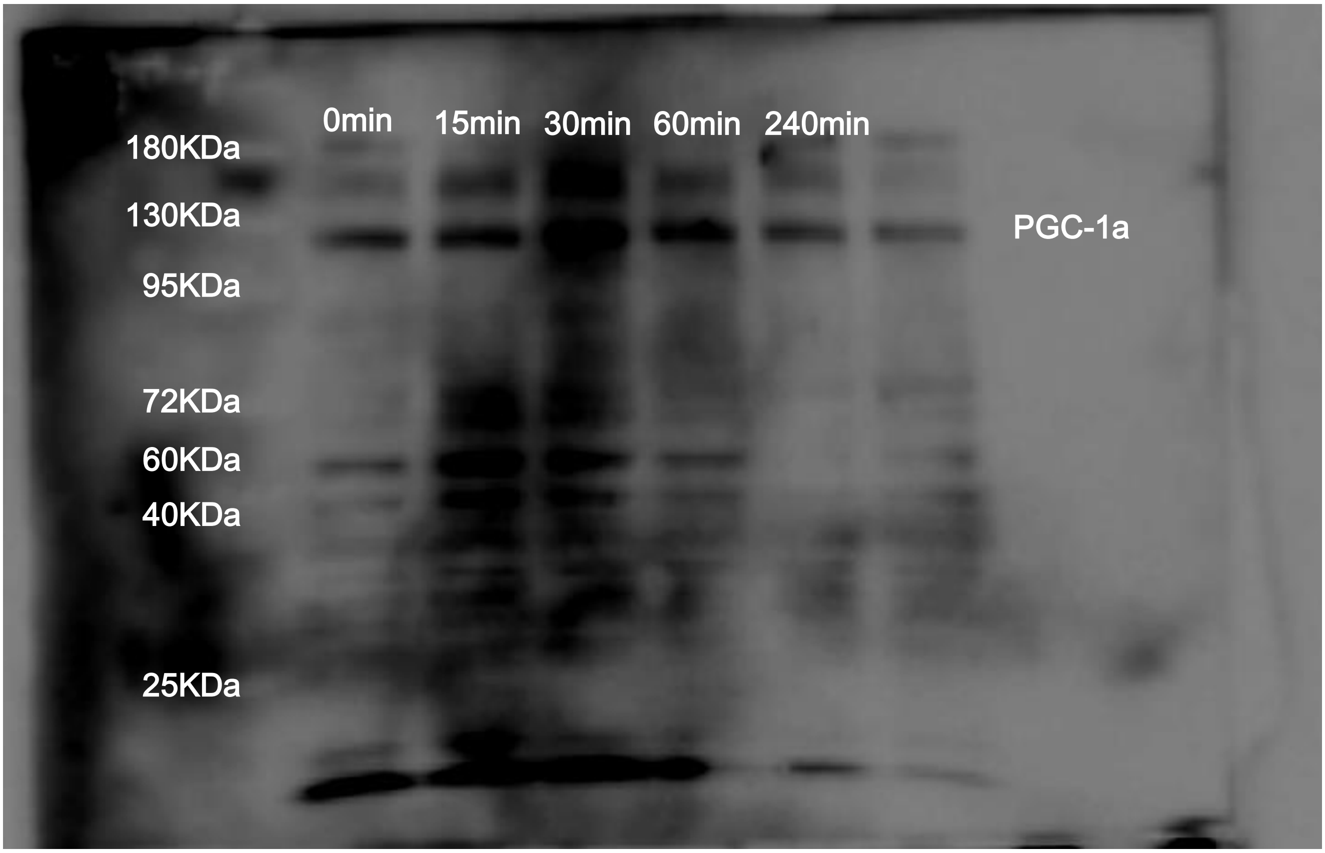

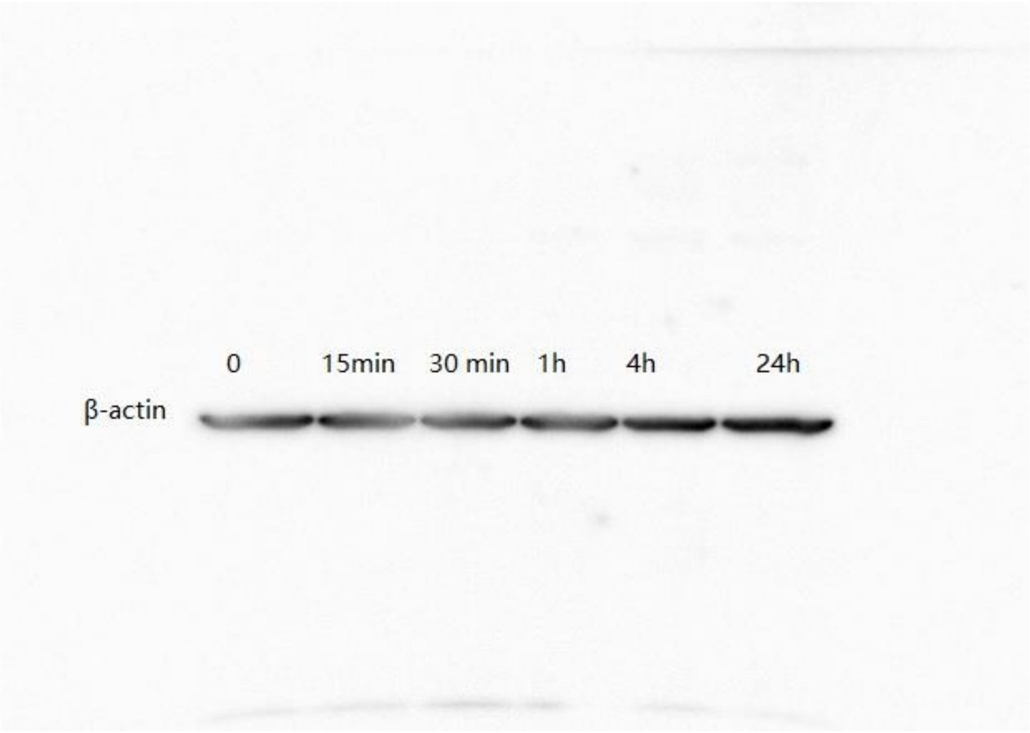

Multiple exposure images for actin of Fig.6b

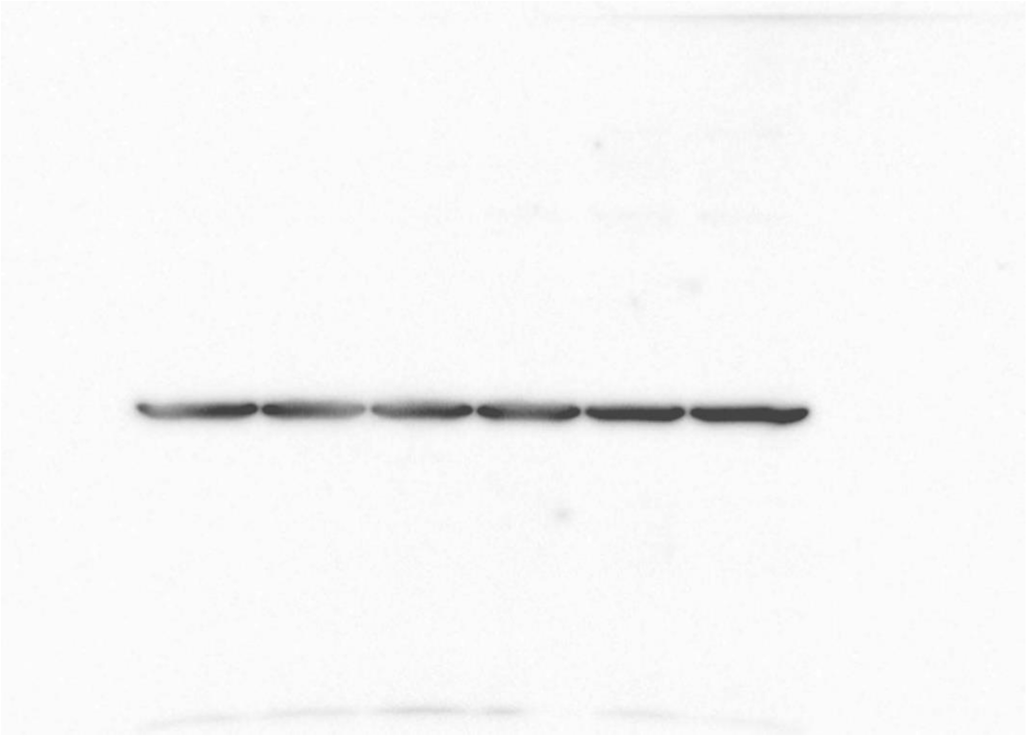

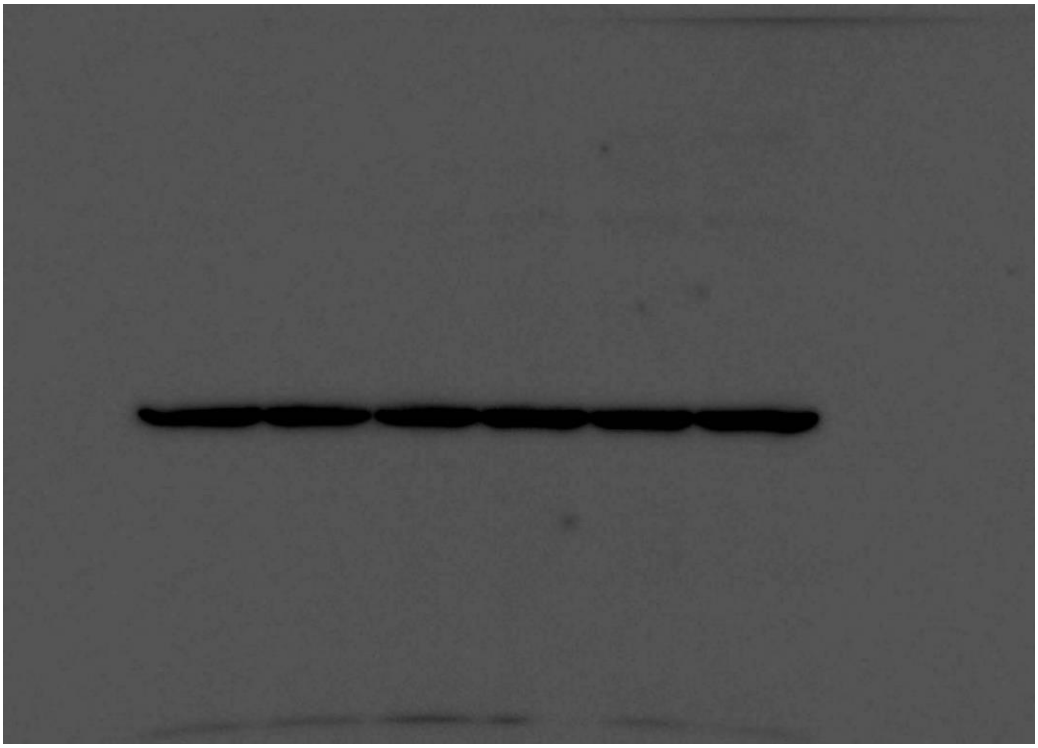

Full unedited gel for Figure 6c

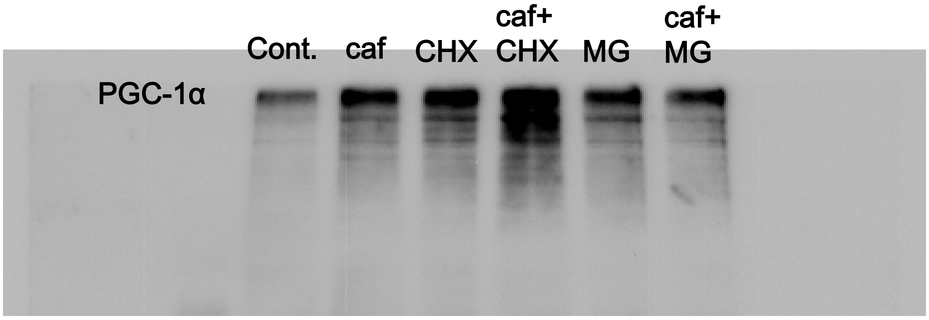

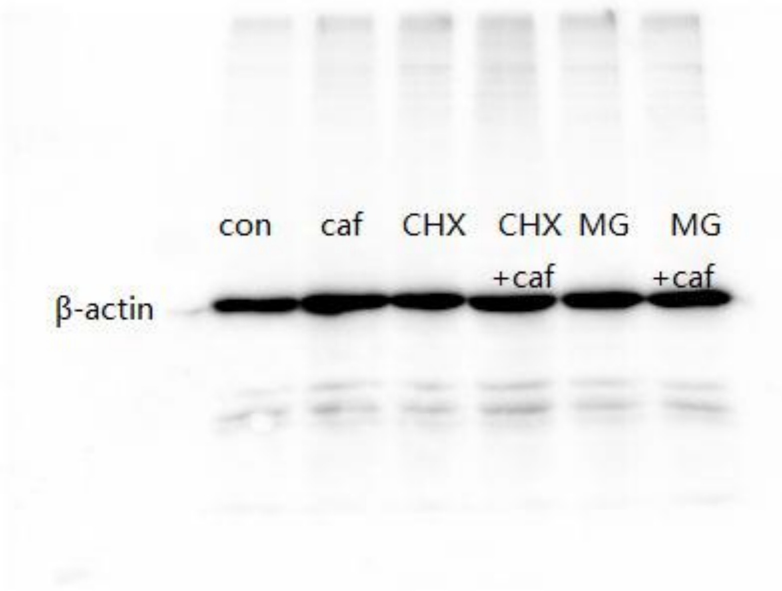

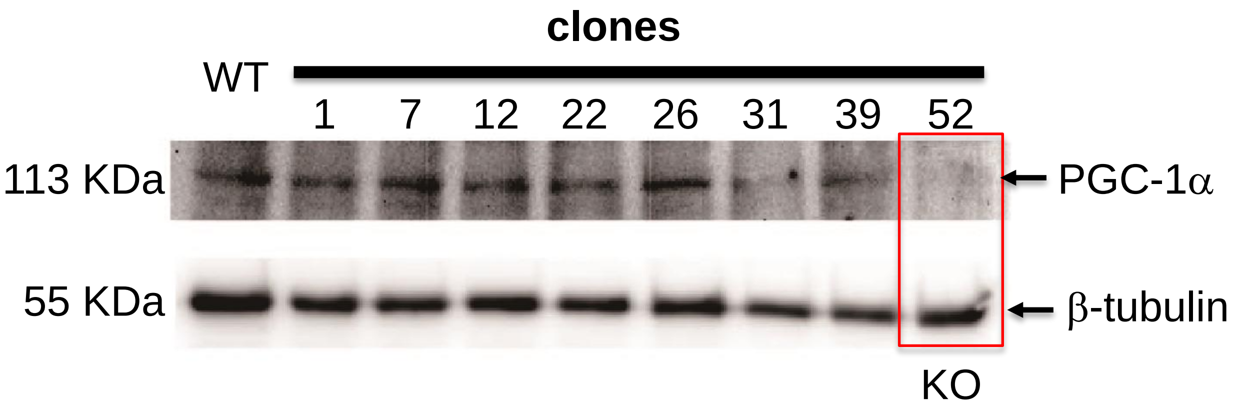

Full unedited gel

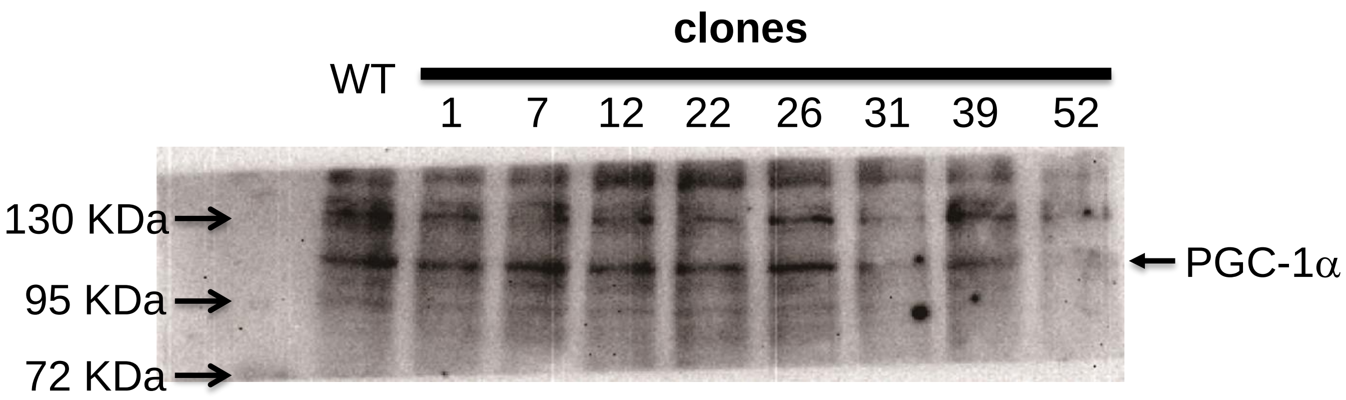

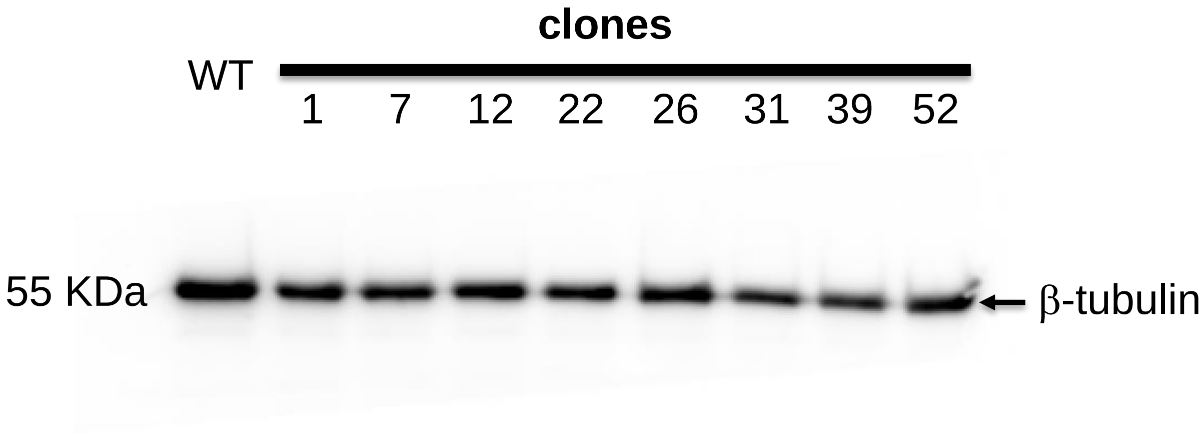

Supplementary Fig. 2 Validation of the anti-PGC-1α antibody specificity

We generated 8 clones from C2C12 cells for possible PGC-1α-deficient genotype by CRISPR/Cas system and obtained one *Pgc1α*$^{/-}$ clone. The anti-PGC-1α antibody used could react with lysates of parental C2C12 cells and non-*Pgc1α*$^{-/-}$ clones but completely not with *Pgc1α*$^{/-}$ clone lysate.

Original tubulin blots for Fig S2 with edges visible

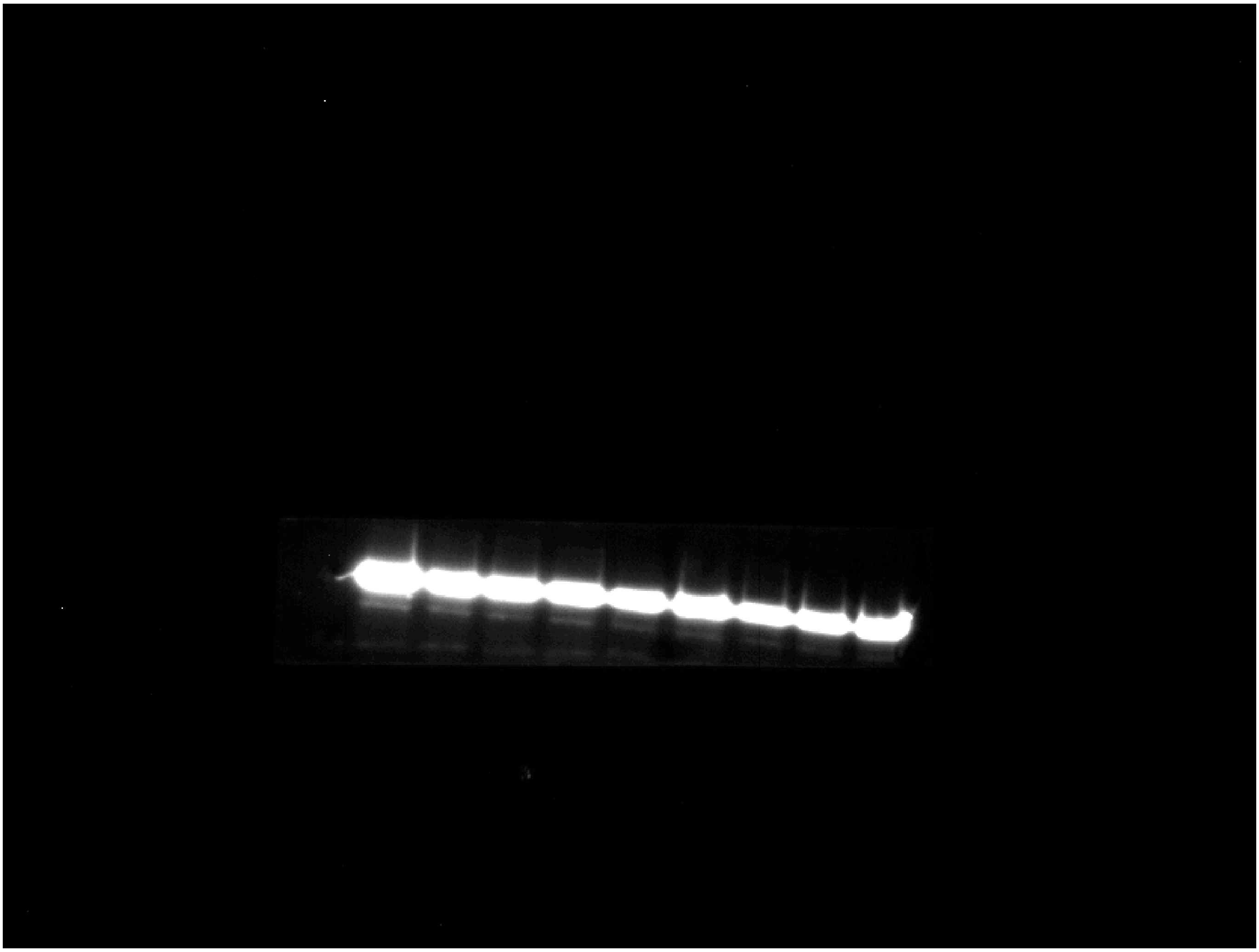

	Monday	Tuesday	Wednesday	Thursday	Friday	Weekend
Week 1	10:00–12:00 Loud noise 15:00–16:00 Restraint 19:00–9:30 （NM） Wet cage	10:00–12:00 Loud noise 14:00–16:00 Stroboscopic illumination 19:00–10:00（NM） Tilted cage	10:00–12:00 Stroboscopic illumination 14:00–15:00 Restraint 16:00–18:00 Loud noise	10:00–11:00 Restraint 14:00–15:00 Stroboscopic illumination 19:00–9:30 （NM） Wet cage	10:00–12:00 Stroboscopic illumination 14:00–16:00 Loud noise 19:00–01:00（NM） Food restriction	Inversion of the light/dark cycle
Week 2	10:00–12:00 Loud noise 14:30—15:30 Restraint 16:00–18:00 Stroboscopic illumination	10:00–12:00 Stroboscopic illumination 14:00–16:00 Loud noise 17:00–9:30(NM) Wet cage	10:00–12:00 Stroboscopic illumination 14:00–16:00 Loud noise 17:00–18:00 Restraint 18:00–9:30（NM） Tilted cage	10:00–11:00 Restraint 14:00–15:00 Stroboscopic illumination 16:00–18:00 Loud noise	10:00–12:00 Stroboscopic illumination 14:00–16:00 Restraint 17:00–10:00（NM） Tilted cage	Inversion of the light/dark cycle
Week 3	10:00–12:00 Stroboscopic Illumination 14:00–16:00 Loud noise 17:00–9:30 （NM） Wet cage	10:00–12:00 Loud noise 15:00–16:00 Restraint 18:00–9:30 （NM） Tilted cage	10:00–12:00 Stroboscopic illumination 14:00–16:00 Loud noise 17:00–9:30 Wet cage	10:00–12:00 Loud noise 14:00–15:00 Restraint	10:00–12:00 Stroboscopic illumination 14:00–16:00 Loud noise 19:00–01:00（NM） Food restriction	Inversion of the light/dark cycle
Week 4	10:00–12:00 Loud noise 14:30–15:30 Restraint 16:00–18:00 Stroboscopic illumination	10:00–12:00 Stroboscopic illumination 14:00–16:00 Loud noise 17:00–9:30（NM） Wet cage	10:00–12:00 Stroboscopic illumination 14:00–16:00 Loud noise 17:00–18:00 Restraint 18:00–9:30（NM） Tilted cage	10:00–11:00 Restraint 14:00–15:00 Stroboscopic illumination 16:00–18:00 Loud noise	10:00–12:00 Stroboscopic illumination 14:00–16:00 Restraint 17:00–10:00（NM） Tilted cage	Inversion of the light/dark cycle
Week 5	10:00–12:00 Stroboscopic Illumination 14:00–16:00 Loud noise 17:00–9:30 （NM） Wet cage	10:00–12:00 Loud noise 15:00–16:00 Restraint 18:00–9:30（NM） Tilted cage	10:00–12:00 Stroboscopic illumination 14:00–16:00 Loud noise 17:00–9:30（NM） Wet cage	10:00–12:00 Loud noise 14:00–15:00 Restraint	Tail suspension test	Inversion of the light/dark cycle
Week 6	10:00–12:00 Loud noise 14:30–15:30 Restraint 16:00–18:00 Stroboscopic illumination	10:00–12:00 Stroboscopic illumination 14:00–16:00 Loud noise 17:00–9:30（NM） Wet cage	10:00–12:00 Stroboscopic illumination 14:00–16:00 Loud noise 17:00–18:00Restraint 18:00–9:30（NM） Tilted cage	Forced swim test	Forced swim test	Inversion of the light/dark cycle

Supplementary Table 1: CMS protocol

Primers	Sequences
Actin	Forward: CGTTGACATCCGTAAAGACC
	Reverse: AACAGTCCGCCTAGAAGCAC
Pgc1α	Forward: TGATGTGAATGACTTGGATACAGACA
	Reverse: GCTCATTGTTGTACTGGTTGGATATG
Kat1	Forward: CGAAGGCTGGAAGGGATCG
	Reverse: GCGGTGAGAAGTCAGGGAA
Kat2	Forward: ATGAATTACTCACGGTTCCTCAC
	Reverse: AACATGCTCGGGTTTGGAGAT
Kat3	Forward: TTCAAAAACGCCAAACGAATCG
	Reverse: GATGACCAAAGCCTCTTGTGT
Kat4	Forward: GGACCTCCAGATCCCATCCT
	Reverse: GGTTTTCCGTTATCATCCCGGTA
Kmo	Forward: TGATGTGTACGAAGCTAGGGA
	Reverse: TCATGGGCACACCTTTGGAAA
Kynu	Forward: TCAAACCCTCCCATTTTGTTGG
	Reverse: CCCCTTGTTTTCGGTGTTATCTT
Ido1	Forward: CAAAGCAATCCCCACTGTATCC
	Reverse: ACAAAGTCACGCATCCTCTTAAA
Ido2	Forward: CCTCATCCCTCCTTCCTTTC
	Reverse: GGAGCAATTGCCTGGTATGT
Tdo1	Forward: AACATGCTCAAGGTGATAGCTC
	Reverse: GAACCGAGAACTGCTGTACCA
Tdo2	Forward: AGGAACATGCTCAAGGTGATAGC
	Reverse: CTGTAGACTCTGGAAGCCTGAT

Supplementary Table 2: Primer sequences
